# Toll-Like Receptors in the Pathogenesis of Alcoholic Liver Disease

**DOI:** 10.1155/2010/710381

**Published:** 2010-08-17

**Authors:** Jan Petrasek, Pranoti Mandrekar, Gyongyi Szabo

**Affiliations:** Department of Medicine, University of Massachusetts Medical School, LRB215, 364 Plantation Street, Worcester, MA 01605, USA

## Abstract

In the multifactorial pathophysiology of alcoholic liver disease (ALD), inflammatory cascade activation plays a central role. Recent studies demonstrated that Toll-like Receptors, the sensors of microbial and endogenous danger signals, are expressed and activated in innate immune cells as well as in parenchymal cells in the liver and thereby contribute to ALD. In this paper, we discuss the importance of gut-derived endotoxin and its recognition by TLR4. The significance of TLR-induced intracellular signaling pathways and cytokine production as well as the contribution of reactive oxygen radicals is evaluated. The contribution of TLR signaling to induction of liver fibrosis and hepatocellular cancer is reviewed in the context of alcohol-induced liver disease.

## 1. Introduction

Alcohol abuse is a leading cause of morbidity and mortality worldwide [1] and alcoholic liver disease (ALD), ranging from steatosis, steatohepatitis to fibrosis and cirrhosis, affects over 10 million Americans [2]. Liver injury mediated by alcohol involves both liver parenchymal and nonparenchymal cells, including resident and recruited immune cells that contribute to liver damage and inflammation [3]. The concept of dysregulated innate immunity as an indispensable component of alcohol-induced liver disease dates back to the observations that patients with ALD have increased antibodies against *Escherichia coli* in plasma [4] and that chronic alcohol administration increases gut-derived endotoxin in the portal circulation, activating resident liver macrophages to produce several proinflammatory cytokines [5, 6]. Recognition of Toll-like receptors (TLR) as the key components involved in activation of the innate immune system enabled a substantial progress in understanding of the mechanisms mediating alcohol-induced liver injury.

## 2. Gut-Derived Bacterial Components Are Critical in the Pathogenesis of ALD

Due to its unique anatomy and blood supply the liver receives blood from the intestine, exposing hepatocytes and cells in the liver sinusoids not only to nutrients but also to gut-derived microbial products. The gut mucosal epithelium serves as an interface between the vast microbiota and internal host tissues [7]. Under normal circumstances, a normal balance of gut barrier function, gut permeability, and equilibrium of commensal and pathogenic microorganisms in the gut lumen is maintained and mostly prevents microbial translocation from the gut [8]. Lipopolysaccharide (LPS, endotoxin), a component of Gram-negative bacterial wall, and other components derived from bacteria in the intestinal microflora normally penetrate the mucosa only in trace amounts, enter the portal circulation, and are cleared by 80%–90% in the liver through uptake by Kupffer cells (resident liver macrophages) and hepatocytes in a manner that prevents cell damage or inflammation [9, 10]. These physiological uptake and detoxification are important for preventing systemic reactions to gut-derived bacterial components.

**Figure 1:**
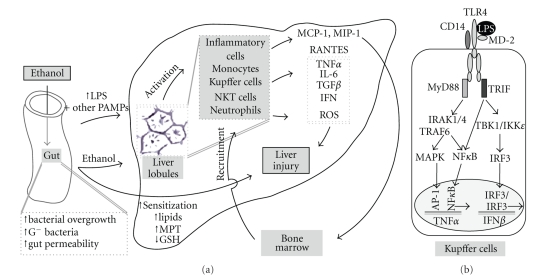
Pathophysiology of alcohol-induced liver injury. (a) Ethanol promotes translocation of LPS and other pathogen-associated molecular patterns (PAMPs) from the gut to the portal vein and to the liver. In the liver, LPS induces activation and recruitment of bone marrow-derived inflammatory cells. Activated bone marrow-derived cells synthesize inflammatory cytokines and reactive oxygen species that induce liver injury. Chronic ethanol per se contributes to sensitization of monocytes/macrophages to LPS and to sensitization of hepatocytes to the cytotoxic effect of inflammatory cytokines. The latter is brought about by accumulation of lipids, opening of mitochondrial permeability transition (MPT) pores, and depletion of glutathione (GSH). (b) In macrophages/Kupffer cells, TLR4 recognizes LPS in cooperation with its coreceptors, CD14 and MD-2. The signal is passed through MyD88-dependent or TRIF-dependent intracellular pathways, which activate various transcription factors, including AP-1, NF*κ*B, and IRF3, and induces proinflammatory cytokine and Type I interferon genes.

Multiple lines of evidence support the hypothesis that gut-derived endotoxin is involved in alcoholic liver injury Figure 1(a). First, it has been shown that excessive intake of alcohol increases gut permeability of normally nonabsorbable substances [11]. Second, intestinal Gram-negative bacteria, as well as blood endotoxin, are increased in acute [12, 13] and chronic [12, 14, 15] alcohol feeding models. Patients with alcoholic fatty liver, alcoholic hepatitis, and alcoholic cirrhosis have 5- to 20-fold increased plasma endotoxin compared to normal subjects [8, 16] although it is unclear whether endotoxemia correlates with the extent of liver dysfunction [17, 18]. Third, intestinal sterilization with antibiotics [19] and displacement of Gram-negative bacteria with *Lactobacillus* treatment [20] prevented alcohol-induced liver injury. The mechanism underlying the disruption of the intestinal barrier appears to be multifactorial [21]. Disruption of tight junctions has been attributed to acetaldehyde [8] and liver-derived inflammatory cytokines, particularly TNF-*α*, that enter the systemic circulation and further disrupt tight junctions, thus perpetuating intestinal barrier dysfunction [22]. Gut permeability may be also increased by ethanol-induction of miR212, a microRNA that downregulates proteins of the zona occludens in intestinal cell culture and that was increased in colonic biopsy samples in patients with ALD [23].

Activation of Kupffer cells has been identified as one of the key elements in the pathogenesis of alcohol-induced liver damage. Kupffer cells are the largest population of tissue macrophages, predominantly distributed in the lumen of hepatic sinusoids, and exhibit endocytic activity against bloodborne materials entering the liver [10, 24]. Triggering of Toll-like receptor signaling drives Kupffer cells to produce inflammatory cytokines and chemokines and to initiate the inflammatory cascade [25]. Indeed, the essential role of Kupffer cells as a central component of the pathomechanism of ALD has been demonstrated in studies in mice and rats that show that inactivation of Kupffer cells with gadolinium chloride or liposomal clodronate can almost fully ameliorate alcohol-induced liver disease [26, 27].

## 3. Toll-Like Receptors Involved in ALD

The innate immune system recognizes conserved pathogen-associated molecular patterns, which are released during bacterial multiplication or when bacteria die or lyse [28], through pattern recognition receptors, including Toll-like receptors (TLRs) [29]. TLR4 recognizes endotoxin from Gram-negative bacteria, and TLR2 is essential for recognition of microbial lipopeptides, while TLR1 and 6 combined with TLR2 distinguish between triacyl- and diacyl-lipopeptides [30]. TLR3 recognizes viral double-stranded RNA, and TLR5 recognizes bacterial flagellin [31, 32]. TLR7 and TLR8 bind viral single-stranded RNA [33], and TLR9 recognizes prokaryotic CpG-rich DNA [34]. Kupffer cells express TLR4, TLR2, TLR3, and TLR9 [35–37], and hepatic stellate cells express TLR2, TLR4, and TLR9 [38, 39]. Liver sinusoidal endothelial cells express TLR4 [40, 41], and primary cultured hepatocytes express mRNA for all Toll-like receptors although they express very low levels of TLR2, TLR3, TLR4, and TLR5 and show weak responses *in vivo* [42, 43].

### 3.1. Role of TLRs in the Pathogenesis of Alcohol-Induced Liver Injury

Activation of Kupffer cells via TLR4-dependent mechanism plays a crucial role in the pathogenesis of alcohol-induced liver injury [6, 19, 44, 45]. LPS, a component of Gram-negative bacteria, is a potent activator of innate immune responses through its binding to the TLR4 complex and comprises three distinct parts: a carbohydrate (O-antigen), the oligosaccharide core region, and a lipid portion (Lipid A). Only the lipid A portion is immunogenic [46]. While TLR4 cannot directly bind LPS, the coreceptors CD14 and MD-2 bind LPS and upon LPS binding activate TLR4. CD14 is a GPI-anchored protein, which also exists in soluble form, and facilitates the transfer of LPS to the TLR4/MD-2 receptor complex that modulates LPS recognition [47]. MD-2 is a soluble protein that noncovalently associates with TLR4 and binds LPS directly to form a complex with LPS in the absence of TLRs [48]. The association between LPS and CD14 is facilitated by LPS-binding protein (LBP), which is a soluble shuttle protein [49]. TLR4, CD14, and LBP are critical in alcohol-induced liver injury. Alcoholic liver injury was prevented in C3H/HeJ mice [50], which have functional mutation in the TLR4 gene and have defective response to bacterial endotoxin [51]. Prevention of alcohol-induced liver inflammation and injury in C3H/HeJ mice was associated with decreased TNF-*α* expression, compared to wild-type mice. Similar protection from alcohol-induced liver injury was observed in mice deficient for LBP [52] and CD14 [53] whereas mice transgenic for human CD14 were hypersensitive to LPS [54]. 

Since disruption of intestinal barrier by ethanol increases permeability for macromolecular substances in general [8], it is likely that other bacterial components, in addition to LPS, are translocated to the portal blood in alcoholics. In particular, bacterial DNA was found in serum and ascites of patients with advanced liver cirrhosis leading to increased cytokine production in peritoneal macrophages [55–57]. Bacterial DNA, which is detected by TLR9, sensitizes the liver to injury induced by LPS via upregulation of TLR4, MD-2 and induction Th1-type immune response in the liver [58, 59]. Hepatic expression of TLR9 was increased in wild-type animals using the Lieber-DeCarli chronic alcohol feeding model, and alcohol feeding sensitized to TLR9 ligand CpG to enhance TNF-*α* production [60]. In patients with alcoholic cirrhosis, purified B cells stimulated with TLR9 ligand CpG *ex vivo* showed significant upregulation of immunoglobulin A, compared to B cells from control individuals [61], suggesting involvement of TLR pathways in alcohol-induced hyperimmunoglobulinemia [61–63]. Also, overexpression of TLR9, TLR4, and TLR2 was associated with impaired neutrophil function in alcoholic liver cirrhosis [64].

In addition to TLR4 and TLR9, increased expression of TLR1, 2, 6, 7, and 8 was observed in wild-type mice using the Lieber-DeCarli chronic alcohol feeding model, and feeding with alcohol resulted in sensitization to liver inflammation and damage because administration of TLR1, 2, 4, 6, 7, 8, and 9 ligands increased expression of TNF-*α* [60]. Interestingly, expression of these TLRs in mice on ethanol diet remained significantly increased in spite of concurrent administration of antibiotics that ameliorated liver injury [60]. Other investigations found that deficiency in TLR2 had no protective effect on alcohol-induced liver injury in a mouse model of chronic ethanol feeding [65] and that hepatic expression of TLR2 or TLR4 mRNA was not changed by chronic alcohol feeding or by acute ethanol administration [66], implying that the increased sensitivity of alcoholic fatty livers to LPS occurs without upregulation of TLR2 or TLR4 genes and may be related to an imbalance of proinflammatory/oxidative and cytoprotective mechanisms.

Taken together, it seems likely that sensitization to TLR ligands in alcohol-induced liver damage is regulated by multiple mechanisms, including those that are directly dependent on gut-derived bacterial components and TLR signaling, but also other mechanisms, such as lipid accumulation in hepatocytes [66, 67], histone acetylation in ethanol-exposed macrophages [68], or activation of Kupffer cells by C3 and C5 components of the complement pathway [69].

#### 3.1.1. MyD88-Independent and Dependent TLR Pathways in ALD

TLR4 is unique among TLRs in its ability to activate two distinct signaling pathways Figure 1(b). One pathway is activated by the adaptors TIRAP (Toll/interleukin-1-receptor- (TIR-) domain-containing adaptor protein) and MyD88, which leads to activation of NF-*κ*B and to the induction of proinflammatory cytokines. The second pathway (MyD88-independent) is activated by the adaptors TRIF and TRAM (TRIF-related adaptor molecule), which activates the TBK/IKK*ε* kinase and interferon regulatory factor 3 (IRF3) to induce Type I IFNs as well as NF-*κ*B activation [70, 71]. The two TLR4-dependent signaling pathways are induced sequentially, and the TRAM-TRIF pathway is only operational from early endosomes following endocytosis of TLR4 [72].

Recent evidence suggests that TLR4 downstream signaling in ALD is mediated predominantly through MyD88-independent pathway, rather than through the MyD88-dependent mechanism. Alcohol feeding with the Lieber-DeCarli diet [73, 74] resulted in significant steatosis and liver damage in MyD88-deficient mice compared to mice on pair-fed diet, and the extent of alcohol-induced changes was comparable in alcohol-fed MyD88-deficient and wild-type mice [65]. The involvement of the MyD88-independent TLR4 signaling pathway was indicated by upregulation of IRF7, an IRF3-inducible gene, in Kupffer cells [75]. In a different study it was reported that mice deficient in TRIF, which is a key TLR4 downstream adaptor in the MyD88-independent pathway, were protected against alcohol-induced liver disease, and it is likely that IRF3, a transcription factor downstream to TLR4/TRIF, binds to the TNF-*α* promoter resulting in induction of TNF-*α* [76]. These findings, together with the observation that mice deficient in IRF3 show protection against alcohol-induced liver injury (Szabo, unpublished data), demonstrate that TLR4-mediated signaling via MyD88-independent pathways is critical in induction of alcoholic liver disease.

#### 3.1.2. Endotoxin Sensing and Loss of TLR Tolerance in ALD

Lipopolysaccharide is the most potent inducer of inflammatory cytokines, particularly TNF-*α*, in monocytes and macrophages. However, when LPS challenge is provided following an initial insult with LPS, induction of TNF-*α* is severely attenuated, a phenomenon called “LPS tolerance.” Recent studies demonstrated that upregulation of negative regulators of TLR signaling play a central role in TLR tolerance [77–79]. Experimental evidence suggests, however, that TLR tolerance can be broken by multiple sequential LPS administration *in vivo* and *in vitro* [80]. When mice were injected with a single dose of LPS, a second LPS challenge failed to induce significant serum TNF-*α* induction compared to the initial dose demonstrating TLR4 tolerance [81]. However, when LPS was given in 3-day intervals for 5 repeated times, the TLR tolerance was lost and serum TNF-*α* levels induced by the last dose of LPS were comparable to TNF-*α* induced by a single LPS administration [9]. The bimodal effects of LPS on TNF*α* production are reminiscent to the opposite modulation of inflammation by acute and prolonged alcohol use. At the cellular and molecular level, acute alcohol administration inhibited while chronic alcohol use increased production of proinflammatory mediators particularly when LPS was used as an inflammatory insult [82]. Increased TNF-*α* production and NF-*κ*B activation were found in monocytes of patients with alcoholic steatohepatitis [83]. 

*In vitro* studies showed that prolonged alcohol exposure of monocytes for 4 days or longer *in vitro* augmented LPS-induced TNF-*α* production compared to alcohol-naïve cells [84]. The involvement of the TLR4 signaling pathway was suggested by increased IRAK-1 phosphorylation, increased IKK kinase activity, increased NF-*κ*B nuclear translocation and DNA transactivation in human monocytes [84]. This upregulation of TLR4 signaling in the presence of diminished expression of IRAK-M in monocytes after prolonged alcohol treatment. Overexpression of IRAK-M prevented the increased LPS-induced TNF-*α* production in chronic alcohol-treated cells suggesting that loss of IRAK-M is likely to contribute to the loss of TLR tolerance in monocytes after prolonged alcohol exposure [84]. 

Previous studies have demonstrated that chronic alcohol use results in increased levels of LPS in the portal and systemic circulation that may mediate or amplify the loss of TLR4 tolerance after chronic alcohol treatment. The role of TLR tolerance and of the loss thereof may deserve further investigation [85].

## 4. Transcription Factors in ALD

The importance of molecular mechanisms culminating in nuclear events leading to activation of a wide array of transcription factors in various liver cell types is widely studied in progression of alcoholic liver injury. These transcription factors bind to the promoter regions in target genes resulting in induction of cytokines, chemokines, and various other mediators including kinases, adaptor proteins, and receptors. Studies using rodent models of alcoholic liver injury show that exposure to chronic alcohol increases expression of genes related to fatty acid synthesis and decreases fatty acid oxidation-related gene expression. Transcription factors such as sterol regulatory binding protein (SREBP) and peroxisome proliferator factor *α* (PPAR*α*) play a pivotal role in fatty acid metabolism and are altered during chronic alcohol consumption. Liver-specific overexpression of SREBP-1*α* and SREBP-1c is observed in alcoholic liver injury leading to increased hepatic triglyceride content [86]. On the other hand PPAR*α* expression and DNA binding activity were decreased in alcoholic livers resulting in decreased fatty acid oxidation [87]. Similar to alterations in transcription factors related to fatty acid metabolism, chronic alcohol-induced inflammatory mediators are also modulated by key transcription factors. The most studied transcription factor is NF*κ*B, and alteration in its DNA binding activity has been observed in livers following chronic alcohol consumption [58] as well as in isolated monocytes/macrophages [88]. Chronic alcohol increased NF*κ*B activity in monocytes and macrophages leading to an upregulation in various inflammatory cytokine and chemokine genes [89, 90]. Another transcription factor modulated by chronic alcohol exposure is AP-1 wherein increased expression and activity were observed in livers of chronic alcohol fed mice [91]. Activation of PPAR*γ*, another transcription factor, was beneficial and prevented chronic alcohol-induced liver injury in mice [92]. While PPAR*γ* is thought to be involved in anti-inflammatory cytokine production, its exact mechanism in alcoholic livers is not known. Furthermore Egr-1, another zinc finger transcription factor, is up-regulated in LPS-stimulated isolated Kupffer cells from chronic alcohol-fed mice and is dependent on ERK activation [93]. Egr-1 knockout mice were protective to alcoholic liver injury, indicating a role for the Egr-1-ERK pathway in the pathogenesis of alcoholic liver injury [94]. Thus, studies so far suggest that transcription factors play an important role in alcoholic liver injury, and future investigations are necessary to determine the complexity of regulation of the target genes in various liver types during alcoholic liver disease.

## 5. Proinflammatory Cytokine Induction in ALD

Alcoholic steatohepatitis is characterized by infiltration of various inflammatory cells in the liver, including monocytes, macrophages, neutrophils, and lymphocytes, which occurs as a consequence of activation of inflammatory mediators induced by TLR signaling [95, 96]. In humans with alcoholic steatohepatitis, serum TNF-*α*, IL-6, and IL-8 levels are increased, and their levels correlate with markers of the acute-phase response, liver function, and clinical outcome [97]. There is also evidence for activation of circulating monocytes in individuals with ALD, based on increased TNF-*α* production and increased NF*κ*B activation [98–100]. 

Induction of TNF-*α* by TLR4 signaling and by reactive oxygen species in Kupffer cells has been identified as a major component in ALD [85, 101, 102]. The effect of TNF-*α* in hepatic inflammation and hepatocyte apoptosis is mediated through TNF receptor TNF-R1 [101]. Binding of TNF-*α* to TNF-R1 activates several signal transduction pathways [103], resulting in the activation transcription factors including NF*κ*B and c-Jun-N-terminal kinase [104] and in activation of proapoptotic Fas-associated death domain [105]. 

Circulating levels of TNF-*α* and TNF-R1 are higher in patients with alcoholic steatohepatitis than in heavy drinkers with inactive cirrhosis, heavy drinkers who do not have liver disease, and individuals with neither alcoholism nor liver disease [45, 83, 106]. High serum levels of TNF-*α* and TNF-R1 correlated with mortality in patients with acute alcoholic hepatitis [106–108]. Hepatic expression of TNF-R1 is enhanced in chronic ethanol consumption [109], and liver injury is substantially reduced when alcohol diet is administered in TNF receptor 1 (TNF-R1)—knockout mice or in rats that have been pretreated with anti-TNF-*α* antibodies or thalidomide, which reduces production of TNF-*α* [110, 111].

Under normal circumstances, hepatocytes are resistant to the proapoptotic effect of TNF-*α*; however, several conditions prime hepatocytes to TNF-*α*-mediated cell death in the setting of chronic alcohol consumption [112–115]. Hepatocytes from rats chronically fed alcohol have increased TNF-*α* induced cytotoxicity associated with mitochondrial permeability transition pore opening [114] and with a profound effect of alcohol on mitochondrial functional integrity [115, 116]. Also, decreased mitochondrial glutathione in alcohol-fed rats [117] or inhibition of hepatic transmethylation reactions by S-adenosylhomocysteine [113] has been shown to sensitize hepatocytes to TNF-*α* mediated cytotoxicity. Moreover, animal models of alcohol-induced liver injury show impaired function of proteasomes that increases hepatocyte sensitivity to TNF-*α*-mediated apoptosis [118]. Interestingly, although upregulation of TNF-R1 is observed in the livers of patients with alcoholic steatohepatitis [109], a recent *in vitro *study showed that free fatty acids sensitized HepG2 cells to TRAIL-mediated apoptosis, but not to cytotoxicity mediated by TNF-*α* [67].

In addition to the metabolic changes involved in sensitization to TNF-*α* cytotoxicity, the net effect of TNF-*α* on hepatocytes is influenced by other cytokines. For example, in mice that are deficient in IL-6, increased production of TNF-*α* induced by partial hepatectomy promotes death of hepatocytes instead of stimulating their proliferation [119]. Similarly, deficiency of IL-10, an anti-inflammatory cytokine inducible by adiponectin [120], exacerbates TNF-*α*-mediated liver injury in mice by alcohol [121]. Conversely, mice that are deficient in interleukin-12 [122], interferon-*γ* [123], or interleukin-18 [124] are protected against TNF-*α*-induced liver damage. The subtle balance between hepatocyte proliferation and apoptosis is also regulated by an autocrine cascade involving the pro-proliferative TGF-*α* and IL-1 receptor antagonist, and the antiproliferative IL-1*β* [125].

## 6. Toll-Like Receptors and Oxidative Stress in ALD

Cellular responses induced by oxidative stress play an important role in innate immune cell activation. Kupffer cells produce reactive oxygen species (ROS) in response to chronic alcohol exposure as well as endotoxin [126]. Interaction of NADPH with TLR4 is involved in LPS-mediated ROS generation and NF*κ*B activation and production of inflammatory cytokines in neutrophils [127] and in human monocytes [128]. Pretreatment of chronic alcohol fed rats with inhibitor of NADPH oxidase diphenyleneiodonium (DPI) normalized ROS production, decreased LPS-induced ERK1/2 phosphorylation, and inhibited increased TNF-*α* production in Kupffer cells [126]. Inhibition of NADPH oxidase prevented steatosis, upregulation of TLR2, 4, 6, and 9 mRNA, and sensitization to respective ligand-induced liver injury [60], indicating a crosstalk between oxidative stress and TLR pathways in ALD. Protection from alcohol-induced liver injury was observed in p47 phox−/− mice, deficient in the main cytosolic component of NADPH oxidase, further supporting the important role of NADPH oxidase in alcohol-induced inflammatory response and liver injury [126].

## 7. TLR Signaling as Target for Therapy of ALD

Recently, a number of different approaches that modulate TLR signaling have been developed. These approaches include modulation of TLR ligand release from the intestine by probiotics [129, 130], activation of TLR signaling by synthetic TLR ligands [131–133], inhibition of TLR activation by small molecule inhibitors [134–136], and interference with cytokines induced by TLR signaling [137–139]. So far, probiotics and anticytokine therapeutic approaches have progressed into clinical trials in patients with ALD [129, 139, 140].

Modulation of intestinal microbiota using probiotics has been shown to reduce bacterial translocation [141, 142], circulating endotoxin levels in animal models [143], and bacterial infection, a marker for bacterial translocation, in patients with liver cirrhosis [144, 145]. Beneficial effects of probiotics have been reported in an animal model of alcohol-induced liver injury [20] and of LPS-induced liver injury [142, 146]. Patients with alcoholic liver cirrhosis treated with *Lactobacillus casei Shirota* three times daily for 4 weeks showed restoration of deranged neutrophil phagocytic capacity, compared to controls [130]. A recent open-label pilot trial showed that a 5-day administration of *Bifidobacterium bifidum* and *Lactobacillus plantarum* in alcohol-addicted psychiatric patients with mild alcoholic hepatitis ameliorated serum markers of liver injury to a significantly higher extent compared to control group treated with abstinence only [129]. These data suggest that modulation of the bowel flora may play a role in the pathogenesis and treatment of ALD and indicate a need for larger and rigorously designed clinical trials to support the use of probiotics in ALD.

While the role of TNF-*α* in the development of ALD has been well characterized [147], clinical investigations of the therapeutic efficacy of antibodies to TNF-*α* (e.g., infliximab) to treat patients with acute alcoholic hepatitis have generated variable results [139, 148]. There is particular concern about off-target effects of completely inhibiting TNF-*α* function. For example, since TNF-*α* is a critical component of immunity, infectious disease is a primary concern during TNF-*α* therapy [139, 149]. Moreover, TNF-*α* is required for normal liver regeneration as hepatocyte proliferation in response to injury is impaired in mice lacking TNF-*α* receptors [150]. Etanercept, a TNF-*α* neutralizing antibody, appeared to increase short-term survival of patients with alcoholic hepatitis in a small pilot study [151] although a subsequent randomized, placebo-controlled trial conducted by the same investigators showed a worse 6-month survival rate in the group treated with etanercept than in the placebo group [152].

## 8. Alcohol: TLR Signaling and Liver Fibrosis

Alcohol-induced liver fibrosis is characterized by excessive deposition of extracellular matrix components due to increased matrix production and decreased matrix degradation [153]. Ethanol contributes to liver fibrosis in several aspects, including the upregulation of collagen transcription in hepatic stellate cells by acetaldehyde or reactive oxygen species from ethanol-exposed hepatocytes [154–157]. Also, phagocytosis of alcohol-induced hepatocyte apoptotic bodies activates hepatic stellate cells and Kupffer cells [158]. 

In addition, cytokines secreted by Kupffer cells activated by alcohol/LPS are of key importance in activation and transformation of hepatic stellate cells and induction of alcoholic liver fibrosis [153, 159, 160]. Recently, it has been shown that the crosstalk between Kupffer cells and hepatic stellate cells involves TLRs on both cell types [161]. Activated hepatic stellate cells express TLR4, CD14, and MD2. Stimulation of activated hepatic stellate cells with LPS resulted in a rapid activation of NF-*κ*B, c-Jun N-terminal kinase and in upregulation of chemokines and adhesion molecules [162].

 Interestingly, stimulation of hepatic stellate cells with LPS alone is not sufficient for their transformation into myofibroblasts. However, pretreatment with LPS strongly enhances response of hepatic stellate cells to TGF-*β*, which is a major profibrogenic cytokine derived predominantly from activated Kupffer cells [163]. The increased sensitivity of LPS-pretreated hepatic stellate cells to TGF-*β* has been linked to a TLR4-dependent downregulation of the TGF-*β* pseudoreceptor Bambi in HSCs, which is a negative regulator of TGF-*β* signaling [163]. Taken together, these findings suggest that LPS influences hepatic fibrosis via TLR4-dependent modification of TGF-*β* signaling in hepatic stellate cells and that hepatic stellate cells represent the primary liver cell compartment integrating inflammatory and fibrogenic pathways [164].

Additional components of the TLR system have been investigated as possible modulators of the fibrogenic process. Upon hepatocyte apoptosis, which is significantly increased in alcoholic liver disease, degradation of nuclear DNA activates immune cells via TLR9 [165]. Activation of TLR9 has been shown to modulate the biology of hepatic stellate cells, including inhibition of cell migration and upregulation of collagen production [38].

## 9. Alcohol: TLR Signaling and Hepatocellular Carcinoma

Alcoholic liver cirrhosis is a premalignant condition with approximately fourfold increase in the risk of hepatocellular carcinoma (HCC) [166]. The five-year cumulative incidence of HCC reaches 8% [167]. In addition, alcohol shows synergy with chronic hepatitis infection [168]. For example, the relative risk of developing HCC was 50-fold higher in heavy drinkers with chronic hepatitis C (HCV) whereas nondrinking HCV patients showed 15-fold increased risk, compared to abstaining controls without HCV [166].

Studies investigating the synergism between alcohol and HCV focused at the structural HCV core [169–171] and the nonstructural NS5A proteins [172, 173]. The HCV core protein causes overproduction of reactive oxygen species [169], induces insulin resistance [171], and inhibits very low density lipoprotein secretion from hepatocytes, contributing to steatosis [170]. However, although HCV core-transgenic mice fed with ethanol for 9 months have shown increased incidence of HCC, the mechanism of synergism between the HCV core protein and ethanol in hepatic carcinogenesis is not known [174].

Recently, the role of TLR4 in the synergism between alcohol and HCV nonstructural protein NS5A in hepatic oncogenesis has been proposed [175]. In a study with NS5A transgenic mice (NS5A Tg), it was reported that NS5A induces TLR4 expression in the liver. NS5A Tg mice developed fulminant hepatitis after administration of a single dose of LPS and showed aggravated alcoholic steatohepatitis after 4-week intragastric ethanol feeding [172]. Importantly, the adjuvant effect of NS5A was blunted in NS5A Tg mice who were deficient in TLR4 or who underwent gut sterilization with antibiotics, indicating the importance of endotoxin and TLR4 signaling in the synergism between alcohol/LPS and NS5A. 

Furthermore, one-fourth of NS5A Tg mice fed Lieber-DeCarli ethanol diet for 12 months developed HCC, in contrast to no tumors found in WT or TLR4^−/−^ NS5A mice, demonstrating that alcohol and NS5A synergistically induce liver tumors through TLR4 signaling [172]. Microarray analysis showed that NS5A Tg mice fed ethanol have increased liver expression of the stem/progenitor cell marker Nanog, which is involved in the genesis of CD133^+^ cancer stem cells. Nanog induction was dependent on NS5A and alcohol and was abrogated in TLR4^−/−^ NS5A Tg mice fed alcohol. Further experiments demonstrated that Nanog is a novel downstream gene of TLR4 signaling. 

Transplantation of p53-deficient hepatic progenitor cells transduced with Nanog or TLR4 resulted in spontaneous tumor development after 80 days or after repetitive LPS injections for 25 weeks, respectively. The tumor incidence caused by TLR4 transduction and LPS injections was reduced by coexpression of short hairpin RNA against Nanog, indicating that Nanog expression is involved in tumor formation and growth in this model [172]. Further experiments showed that Nanog-positive cancer stem cells did not upregulate TGF-*β* signaling after TLR4 activation [173]. Defective TGF-*β* pathway leads to spontaneous development of HCC [176].

Taken together, the recent data [172, 173] suggest that alcohol and HCV NS5A induce synergistic tumor development via induction and activation of TLR4 in mice and that this synergism involves the stem cell marker Nanog, which is a TLR4-downstream regulated gene. These findings indicate that inhibition of TLR4 signaling may provide a therapeutic option for HCV-associated liver tumors.

## 10. Conclusion

In conclusion, there is clear evidence that alcohol consumption leads to increased intestinal permeability and endotoxemia, which results in activation of innate immunity via TLR4 signaling. Recent studies have contributed to the dissection of molecular mechanisms of TLR4 signaling in ALD, indicating the indispensable role of MyD88-independent pathway in mediating the effects of gut-derived endotoxin in ALD and suggesting the role of other TLRs in modulation of alcohol-induced liver injury. Moreover, novel data provide insight into the mechanisms of prolonged alcohol exposure on TLR4-induced inflammation and loss of LPS tolerance and the interplay between proinflammatory and anti-inflammatory cytokines mediating TLR-induced cytotoxicity. Further studies are needed to evaluate crosstalk between liver parenchymal and nonparenchymal cells. Understanding the cell-specific role of TLR signaling in ALD will further provide new insights into the pathogenesis of ALD and will reveal new targets for therapeutic intervention.
